# Epidemiologic Characteristics of Imported and Domestic Chikungunya Cases in Taiwan: A 13-Year Retrospective Study

**DOI:** 10.3390/ijerph17103615

**Published:** 2020-05-21

**Authors:** Yu-Ching Chou, Chi-Jeng Hsieh, Chun-An Cheng, Ding-Chung Wu, Wen-Chih Wu, Fu-Huang Lin, Chia-Peng Yu

**Affiliations:** 1 School of Public Health, National Defense Medical Center, Taipei 114, Taiwan; trishow@mail.ndmctsgh.edu.tw (Y.-C.C.); doctor0317@yahoo.com.tw (W.-C.W.); noldling@ms10.hinet.net (F.-H.L.); 2 Department of Health Care Administration, Oriental Institute of Technology, New Taipei City 220, Taiwan; fl004@mail.oit.edu.tw; 3 Department of Neurology, Tri-Service General Hospital, National Defense Medical Center, Taipei 114, Taiwan; cca@ndmctsgh.edu.tw; 4 Department of Medical Records, Tri-Service General Hospital, National Defense Medical Center, Taipei 114, Taiwan; wuper@mail.ndmctsgh.edu.tw; 5 Department of Surgery, Suao and Yuanshan Branches of Taipei Veterans General Hospital, Yilan County 270, Taiwan

**Keywords:** chikungunya, surveillance, domestic, imported, epidemiology

## Abstract

**Background:** Chikungunya fever is caused by the chikungunya virus. Numerous factors affect the risk of chikungunya transmission. This study explored the epidemiological characteristics, differences, and trends in domestic and imported cases of chikungunya fever in Taiwan in terms of patient sex, age, month of confirmation, and area of residence from 2007 to 2019. **Methods:** Public annual chikungunya data from Taiwan’s Centers for Disease Control (CDC) were analyzed. In total, 21 confirmed domestic and 198 imported cases of chikungunya were reported. Of the domestic cases, one was sporadic and reported in July 2019, and 20 were attributed to a cluster event during August and September 2019. **Results:** In a comparison between domestic and imported cases reported from July to October 2019, differences in sex were nonsignificant (*p* = 0.555), whereas significant differences were observed for age (*p* < 0.001), month of confirmation (*p* = 0.005), and place of residence (*p* = 0.001). An age of 69–69 years (odds ratio (OR) = 6.66, 95% confidence interval (95%CI) = 2.15–20.65), month of confirmation of September (OR = 5.25, 95%CI = 1.89–14.61) and place of residence of New Taipei City (OR = 48.70, 95%CI = 6.17–384.44) were identified as potential risk factors. Additionally, domestic cases in August and September 2019 increased in proportion to the increase in imported cases during July and August 2019. Increased domestic patients may have been caused by the domestic mosquitoes that transmitted the virus by biting the imported patients to Taiwan. This is the first report comparing domestic and imported cases of chikungunya from surveillance data from the Taiwan CDC from 2007 to 2019. **Conclusion:** This study highlights the importance of longitudinal and geographically extended studies to understand the implications of zoonotic disease transmission on Taiwan’s population. Critical data were identified to inform future surveillance and research efforts in Taiwan.

## 1. Introduction

Frequent international interactions (e.g., total international tourist arrivals grew 5% in 2018 to reach the 1.4 billion mark, according to data from the World Tourism Organization), global climate change, and changes in living environments have resulted in the rapid spread of various emerging and recurrent infectious diseases worldwide—particularly vector-borne diseases—and their increased frequency. Chikungunya is an acute infectious disease transmitted by vector mosquitoes [1]. After infectious patients with chikungunya virus enter the community, vector mosquitoes in residential areas can cause a chikungunya epidemic [2].

The chikungunya virus is a positive-sense single-stranded RNA virus classified into the genus *Alphavirus* of the *Togaviridae* family [3]. It has three genotypes, namely the West African, East/Central/South African (ECSA), and Asian genotypes [4]. Since 2005, the ECSA genotype has caused epidemics that have spread from East Africa to Indian Ocean islands, India, and Southeast Asia, and it has caused indigenous clusters for the first time in many regions, including the Middle East and Europe. Since 2013, the Asian genotype has caused major clusters for the first time in the Americas and the Pacific islands [5].

Chikungunya epidemics have been mainly distributed in tropical and subtropical regions of Africa, Asia, and the Americas [6]. Chikungunya spread in Tanzania, East Africa from 1952 to 1953 [7] and in various other African and Asian regions between 1960 and 1982; a large-scale outbreak occurred in the Democratic Republic of the Congo [8] in 1999–2000. Since 2004, it has been spread continually in Sri Lanka, the Maldives, Thailand, Malaysia, Singapore, India, Saudi Arabia, New Guinea, and islands in the Indian Ocean including Madagascar, the Seychelles, Comoros, Mayotte, and Réunion [9]. A severe outbreak occurred in the Gabonese Republic in 2007. In the same year, Italy was the first European country to which the disease spread; 205 confirmed cases were reported [10]. An outbreak occurred in Singapore in 2008. In October 2013, Micronesia became the first West Pacific island to have locally infected cases. In December of the same year, an outbreak took place in the Caribbean, marking the first locally infected cases in the Americas [11]. In 2014, an outbreak started in the Pacific islands, including the Marshall Islands and Cook Islands. Chikungunya has spread to more than 60 countries in Asia, Africa, Europe, and the Americas [12]. According to public data on the online platform of the Pan American Health Organization [13,14], 693,489 suspected cases of chikungunya were reported in the Americas in 2015, of which 37,480 were confirmed cases; the number of confirmed cases increased to 152,769 the following year. Chikungunya spread to Argentina for the first time in March 2016. In 2017, the number of confirmed cases in the Americas totaled 123,087, 98% of which occurred in Brazil; this indicated a high risk of chikungunya spread in the Americas. Since the March 2019 outbreak, Ethiopia has reported 20,000 cumulative confirmed cases. Infection occurs through bites by vector mosquitoes, mainly *Aedes aegypti* and *Aedes albopictus* [15]. Individuals are infected by bites from mosquitoes carrying the chikungunya virus.

The clinical symptoms of chikungunya are fever, joint pain or arthritis, headache, nausea, fatigue, and muscle pain, and approximately half of patients develop a rash that lasts approximately 3–7 days [16]. Most patients recover within 1 week and are unlikely to die. “Chikungunya” originates from a verb in the Kimakonde language meaning “to become contorted”; this refers to the stooped posture of patients with joint pain. Higher-risk groups include newborns, people older than 65 years, and those with high blood pressure, diabetes, or cardiovascular disease [17].

Because Taiwan is an island, most infectious diseases (including vector-borne disease) spread to Taiwan via traffic vehicles entering its seaports and airports. Since chikungunya was declared a category-2 infectious disease in the Communicable Disease Control Act in October 2007 and the beginning of the chikungunya network surveillance report in December 2007, Taiwan has had an average of 16 confirmed imported and domestic cases per year [18]. Additionally, to the authors’ knowledge, no research has provided longitudinal epidemiological data on chikungunya in Taiwan. Therefore, the present study employed data from the Taiwan Centers for Disease Control’s (CDC) Taiwan National Infectious Disease Statistics System (TNIDSS) to analyze the epidemiological characteristics, differences, and trends among imported and domestic cases of chikungunya in Taiwan between 2007 and 2019.

## 2. Materials and Methods

### 2.1. Ethical Policy

This study did not require ethical approval because it involved information freely available in the public domain. Analysis was performed on open-source data sets in which data are properly anonymized.

### 2.2. Data Source

This study employed the TNIDSS public network database [18], which included all notifiable communicable diseases in categories 1–5 (e.g., chikungunya) specified by the Communicable Disease Control Act. The provision of these data has maintained the transparency and currency of Taiwan’s disease information. To ensure information security and prevent personal information leaks, the TNIDSS does not store personal information and stores only secondary statistics. These statistics enable the public, academics, and the press to access current information on chikungunya in Taiwan at any time. Chikungunya was categorized as a category-2 notifiable communicable disease by the central competent authority of Taiwan in 2007. The present study collected the following data from the TNIDSS: date when confirmed cases of chikungunya were reported to the health department, date of symptom onset, date of case confirmation, numbers of confirmed domestic and imported cases, and patients’ region/location, age, and sex. The database does not contain patients’ medical histories, signs and symptoms.

### 2.3. Definition of Cluster Infections

A cluster infection is an aggregation of cases grouped in terms of location and time that are suspected to be greater than the number expected, even though the expected number may not be known.

### 2.4. Definition of Confirmed Cases (Included Laboratory Examination)

In Taiwan, chikungunya is a notifiable communicable disease, which is a disease classification determined by the central government in the Communicable Disease Act. For such diseases, doctors or medical institutions must report confirmed cases to the competent health authority and enact measures such as treatment or event isolation pursuant to the act. Notifiable communicable diseases are typically characterized by rapid spread, severe symptoms, or high mortality rates. Therefore, when doctors encounter patients suspected to have chikungunya, they must report these patients to the competent health authority within 24 h. Suspected patients with chikungunya exhibit associated clinical conditions (i.e., acute fever ≥38 °C and severe arthritis or arthralgia that cannot be explained by a medical diagnosis) as well as one of two epidemiological conditions (i.e., the appearance of confirmed cases of chikungunya in an area near patients’ residences or areas they regularly visit or a travel history to places with chikungunya). Patients then receive a test at the hospital; test results are deemed positive if they exhibit one of the following conditions: (1) the separated clinical specimen (blood) contains the chikungunya virus, indicated by nucleic acid testing yielding a positive result; (2) acute-phase serum (or serum collected the first time) tests positive for one of two specific antibodies (i.e., IgM or IgG) for the chikungunya virus; and (3) the paired serum (serum in the recovery and acute phases) exhibits seroconversion for one of two specific antibodies (IgM or IgG) or an increase in antibody level by ≥4 times. The positive results of the PCR viral tests could also represent non-live virus. In this study, in addition to PCR positive results, other epidemiological information or clinical characteristics could be considered in the definition of positive patients. According to the Taiwan CDC, a confirmed case of chikungunya must meet one of the above criteria.

### 2.5. Data Analysis and Statistics

According to the research framework, this was a retrospective historical study on all imported and domestic cases of chikungunya since 2007. On the basis of the TNIDSS, the numbers of imported and domestic confirmed cases of chikungunya from January 2007 to October 2019 were determined, and the distribution, differences, and results of epidemiological characteristics (i.e., sex, age, month and date of confirmation, and place of residence) were consolidated. Descriptive data are expressed as means and summary statistics. Categorical variables were compared using chi-square tests, which were performed using SPSS (IBM SPSS Statistics 21; Asia Analytics Taiwan Ltd., Taipei, Taiwan). Chi-square tests were two-sided with an α level of 0.05. Values for which *p* < 0.05 were considered statistically significant.

## 3. Results

**Figure 1:**
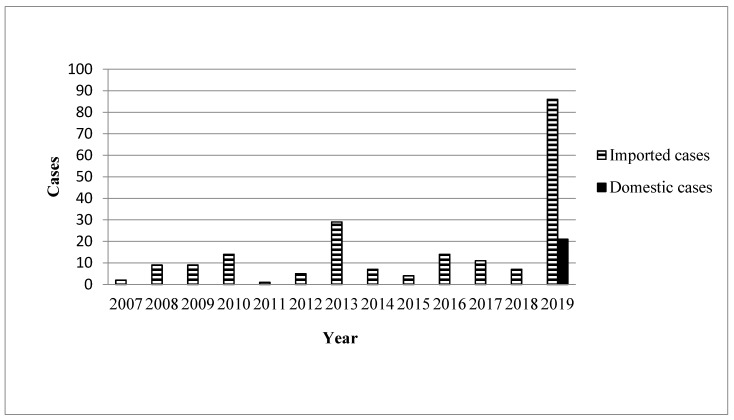
Yearly distribution of chikungunya associated with domestic and imported patients during 2007 and 2019.

**Figure 2:**
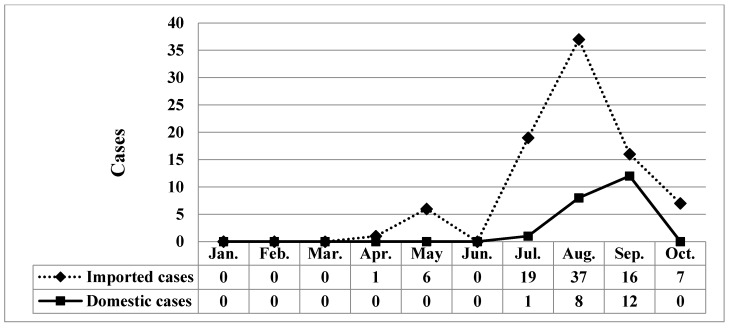
Monthly distribution of chikungunya associated with domestic and imported patients during 2019.

**Figure 3:**
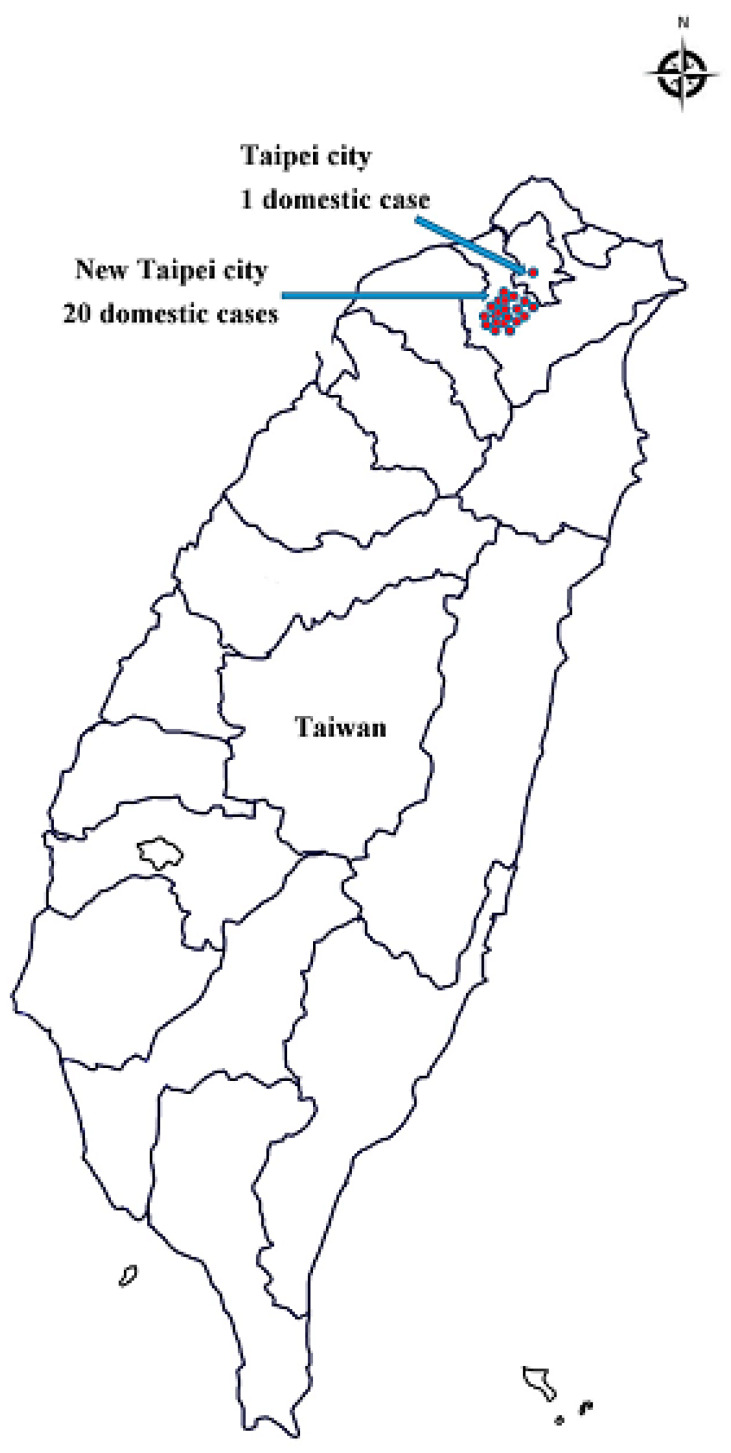
Regional distribution of chikungunya associated with domestic confirmed cases, Taiwan, 2007–2019.

During the study period (January 2007–October 2019), 219 confirmed cases were reported (Figure 1), of which 86 imported cases and 21 domestic cases were reported between January 2019 and October 2019 (Figure 2). Figure 3 illustrates the regional distribution of domestic confirmed cases of chikungunya in Taiwan from 2007 to 2019.

A total of 119 imported cases were reported from January 2007 to June 2019. Among the 100 cases reported between July 2019 and October 2019, 79 were imported, and 21 were domestic; 34 patients were male, and 66 were female. Additionally, five patients were aged <20 years, 74 were aged 20–59 years, and 21 were aged >60 years. In 2019, 20 cases occurred in July, 45 occurred in August, 28 occurred in September, and seven occurred in October. Taipei City had 21 cases, New Taipei City had 43 cases, and other areas had 36 cases (Table 1). In the comparison between domestic and imported cases of chikungunya between July and October 2019, differences in sex were nonsignificant (*p* = 0.555), whereas a significant difference in age (*p* < 0.001), month of confirmation (*p* = 0.005), and place of residence (*p* = 0.001; Table 1) were observed.

## 4. Discussion

Because of global climate change and the increased travel between continents, tropical diseases including chikungunya are more likely to spread in Taiwan. Humans face new challenges in the control of these diseases. This study analyzed publicly available annual summary data of reported imported and domestic chikungunya cases from 2007 to 2019 published by the Taiwan CDC. All 112 chikungunya cases reported during and before 2018 were imported. The first domestic case was reported in July 2019 in a woman between 20 and 30 years old living in Tu-Cheng District, New Taipei City [19]. The patient’s activity area encompassed her home and an area with a high population of foreign workers, and she had no history of travel abroad. The patient reported having been bitten by mosquitoes during her activities in Tu-Cheng District. This suggests that undetected imported chikungunya cases were in the area. A few days after biting a person, a mosquito can carry the virus and cause more domestic infections. On 21 July 2019, the patient presented symptoms including fever, joint pain, headache, and rash. When the patient reported to the hospital, the doctor suspected dengue fever or measles; although testing excluded a suspected infection, chikungunya was confirmed in a routine test. Currently, the patient’s symptoms have improved, and she was isolated at home until the epidemic warning ended. Those who had contact with the patient have not exhibited any symptoms of chikungunya.

Since July 2019, domestic cases of chikungunya have been observed in Taiwan, including one single case in July and 20 cluster cases in August and September. Between January 2019 and October 2019, imported cases reached a cumulative total of 86. Most of the imported cases originated in Myanmar. The disease situation in Thailand has worsened and entered a rapid-spread phase, and India has witnessed a cumulative total of 40,000 suspected cases. Therefore, the Taiwan CDC issued a level-2 (alert) travel notice for Myanmar and level-1 (watch) travel notices for Thailand, India, and the Maldives on 22 October, 2019 [20]. Hot zones for chikungunya are mainly distributed in tropical and subtropical areas of the Northern Hemisphere, in which relatively high temperatures and numerous travelers are the main causes of disease spread. In other words, airlines contribute substantially to virus transmission, and busy airports are breeding grounds for the virus. Therefore, the government should provide disease information and establish control measures for inbound travelers from disease hot zones to improve the effectiveness of epidemic control. This study compared the domestic and imported cases of chikungunya from July 2019 to October 2019 and revealed a nonsignificant difference in patient sex. Specifically, sex was not a risk factor for chikungunya; however, the attack rate was slightly higher in women (71.4% for domestic cases and 64.6% for imported cases) than in men. Patient age exhibited a significant difference. Patients with domestic cases were mainly 60–69 years old (attack rate = 42.9%), whereas those with imported cases were predominantly 20–29 years old (attack rate = 30.4%). The older population accounted for a larger proportion of domestic confirmed cases, probably because older adults visited mountain areas that have vector mosquitoes (infected areas) more frequently than young people did, and they tended to underestimate the risk of the epidemic. A significant difference also emerged in the month of confirmation; specifically, the number of domestic confirmed cases increased in August and September proportionally to the increase in imported cases confirmed in July and August. This proportional increase may have occurred because patients with imported cases were bitten by noninfected vector mosquitoes that spread the disease, thereby indirectly increasing the number of domestic cases. A significant difference was also observed in the patients’ places of residence. Surprisingly, although Taipei City and New Taipei City exhibited similar numbers of imported cases, the proportion of domestic cases in New Taipei City to the total number of domestic cases of the two cities combined (95%, 20/21) was 19 times that of domestic cases in Taipei City (5%, 1/21). Such a high ratio may have been caused by patients who were infected in clusters in New Taipei City regularly visiting noncentral metropolitan areas near mountains; thus, they had a higher risk of being bitten by vector mosquitoes. In contrast, Taipei City is a metropolitan center in which residents were probably more aware of the disease and tended to maintain neighborhood cleanliness, contributing to the lower number of confirmed cases. Overall, differences between imported and domestic cases in terms of patient age, month of confirmation and place of residence may be risk factors for chikungunya in the Taiwanese population.

Research outside Taiwan has demonstrated that hot environments are associated with rapid disease transmission by vector mosquitoes [21]. For example, the time required for vector mosquitoes to transmit the dengue virus to uninfected people changes with temperature [22]: transmission requires 25 days at 26 °C but only 7 days at 32 °C. Because of the increasing temperatures every decade over the past 40 years, the United Nations’ World Meteorological Organization stated in its annual assessment report [23] in late 2019 that climate change has progressed beyond the human capacity to adapt. Global temperatures since 2019 have exceeded those before the industrial revolution by 1.1 °C, and this decade will be the hottest in history. According to Taiwan’s Central Weather Bureau [24], Taiwan’s average temperature in 2019 was 24.56 °C, which was higher than that of 2018 by 0.34 °C and the highest since 1947. Therefore, the first cluster of confirmed cases of chikungunya in the fall of 2019 was probably caused by the increased temperatures during this year which increased the number of vector mosquitoes and subsequently the number and frequency of bites. The increase in temperature also accelerated viral replication in vector mosquitoes and expanded the spread of disease. In other words, Taiwan faces climate change and a climate emergency, both of which require an immediate response. Because of global warming, fall and winter have become warm; this has contributed to the spread of vector-borne disease, posing a great threat to public health. The government should plan public-health policies in advance and thoroughly implement preventive measures to safeguard the health of citizens.

Zoonoses refer to infectious diseases that are transmissible between animals and humans. Such transmission occurs either directly or indirectly through vectors between humans and animals to transport pathogens from one organism to another. Types of pathogens include viruses, bacteria, mold, parasites, and protozoa. Their modes of transmission are direct contact with or inhalation of pathogens, consumption of food or water containing pathogens, and vector bites [25,26]. Chikungunya is a zoonosis spread by vector mosquitoes (*Aedes aegypti* and *Aedes albopictus*) that bite natural hosts. The chikungunya virus is maintained in nature in both urban and sylvatic cycles involving mosquito vectors and human or vertebrate animal hosts [27]. A study indicated that the unprecedented destruction of natural barriers between nonhuman primates and humans has increased health risks for a large population, including people living in urban areas [28]. Additionally, the chikungunya virus is an RNA virus with a high mutation rate of up to one million times higher than that of their hosts, and these high rates are associated with enhanced virulence and evolvability, which are beneficial traits for viruses [29]. Population growth as well as improper and excessive land development in Taiwan have damaged natural spaces and disturbed ecosystems, thereby increasing human exposure to wild animals and vector insects. Therefore, residents of cities (or the countryside) in Taiwan typically live in the same geographical areas as macaques living in mountains. It was unknown whether any study has described chikungunya infection in primates in Taiwan. Such a geographical area of modern urban living space is also a hot zone of viral spread by arthropod vectors. In particular, international studies have reported that the chikungunya [9] and dengue viruses [30], which are spread by vectors, are often transmitted between humans and monkeys. Such cross-species infection poses a severe threat to human health. According to a Taiwan CDC report [31], the 20 chikungunya cluster cases in Taiwan overlapped considerably with imported cases from Myanmar in terms of the patients’ neighborhoods of residence and regularly visited places. Patients with imported cases from Myanmar lived only hundreds of meters away from those with domestic cases, and patients with both imported and domestic cases has visited mountain trails in their neighborhoods (risk areas) during the incubation period. Among water-holding containers along the mountain trails tested by a health and environmental protection specialist, 24.6% tested positive for *Aedes albopictus*, and they had a level-6 container index, indicating an extremely high risk of vector mosquito transmission. Laboratory results indicated that the DNA sequence of the virus in an imported case from Myanmar had high affinity to that of the domestic cases. Therefore, this study inferred that the genotype of the virus strain that infected the chikungunya cluster cases in 2019 originated in Myanmar. A study also indicated that two genotypes of chikungunya virus strains were imported to Taiwan between 2006 and 2014, namely ECSA and Asian [32]. The ECSA genotype was the most commonly imported genotype of the chikungunya virus before 2010; strains with this genotype originated mainly in Bangladesh, Malaysia, India, and Thailand. The Asian genotype was most common after 2011; strains with this genotype were mainly from Indonesia, the Philippines, and Singapore. This serves as a reminder for Taiwan’s vulnerable public-health system that, in addition to understanding pathogens, Taiwan must adopt comprehensive disease control measures, enhance environmental protection, and prevent the breeding of vector mosquitoes to effectively prevent the spread of chikungunya and protect the public from this health crisis.

Because the government regards the epidemic as a war, this study referred to the Taiwan CDC’s promotion of epidemic prevention [33] and revealed numerous breeding sites in mountainous areas. However, epidemic control in those areas is difficult. Compared with the dengue virus, the chikungunya virus requires less time to replicate in the body; thus, it has a higher transmission speed than the dengue virus does. Vector mosquitoes of chikungunya are not selective regarding bite targets, implying that, after the chikungunya virus enters a community that contains breeding sites for vector mosquitoes, chikungunya spread is possible. Therefore, people should ensure that breeding sites for vector mosquitoes are located away from living spaces. This study proposed the following recommendations to prevent chikungunya: (1) measures should be enacted to prevent mosquito bites among people in areas with chikungunya spread. These people may reside in buildings with installed window screens or air conditioning. During outdoor activities, they should apply mosquito repellents that are certified by the competent authority to contain N,N-diethyl-meta-toluamide or picaridin [34]. If they plan to remain outdoors for an extended period, they should wear long-sleeved tops and pants on which mosquito repellent should be applied to enhance protection. (2) Preventive measures can be enacted at home and during outdoor activities. All vases and water-holding containers, particularly their inner surfaces, should be washed weekly. To prevent vector mosquitoes from breeding in stagnant water, all indoor vases and containers not in use should be stored upside down. All waste tires and outdoor water-holding containers should be removed immediately. Additionally, epidemic-control specialists and public-health experts may conduct epidemic-control interventions as well as promote and communicate relevant information to medical practitioners and the public through media in a timely manner to improve people’s understanding and awareness of chikungunya. Such collaboration among health and epidemic-control agencies, clinical doctors at medical institutions, and the public may facilitate effective control of the spread.

In addition to the aforementioned preventive measures to control infectious disease, vaccine development is an epidemic prevention strategy to induce the human body’s immunity to external pathogens and achieve community immunity. Although the chikungunya virus is not associated with a high mortality rate, its severe and chronic symptoms have motivated experts to rapidly develop a vaccine. Vaccine development for the chikungunya virus began in 1960, when large-scale spread occurred in Thailand. Regarding progress until 2018, Reisinger and colleagues assessed the immunogenicity, tolerability, and safety of a live attenuated measles-vectored vaccine that expresses the chikungunya virus structural protein in a double-blind, randomized, placebo-controlled, and active-controlled phase-2 trial. The results revealed that the vaccine has a strong safety and immunogenicity profile, and plans for a phase 3 study are in preparation [35] Problems remain for this vaccine (e.g., it is unclear whether it is feasible for the genetics of different ethnicities). However, an expert of the US CDC argued that “the report of a successful chikungunya virus vaccine phase 2 clinical trial that might lead to a protective, licensed product in the future is a clear win for all those still at risk of infection by this highly debilitating arbovirus” [36]. Therefore, an effective chikungunya vaccine should be developed to prevent vector-borne diseases and safeguard human health.

Apart from chikungunya, the most severe and deadly vector-borne disease that has spread widely and caused cluster infections in Taiwan is dengue fever, which has particularly affected Southern Taiwan. Both dengue fever and chikungunya are transmitted by the same types of vector mosquitoes. Dengue fever has been detected widely throughout Southern Taiwan and sporadically in the north [37]. Warmer temperatures favor its survival, and new cases are less likely to occur in winter [37]. However, if global temperatures increase, vector mosquitoes may spread further north in Taiwan, and small clusters of cases may appear in the north [38]. Chikungunya and dengue fever have similar incubation periods (from 1–2 days before symptom onset to 5 days after the symptom onset) and viremia periods (3–8 days) [39]. Thus, they are difficult to distinguish. The clinical conditions of patients contracting these two diseases typically become less severe over 1–2 weeks. For patients who have traveled to areas with chikungunya spread and have suspected dengue fever but have tested negative to the dengue NS1 test, the clinical doctor should report them as having a suspected case of chikungunya to more effectively identify people with chikungunya before symptom onset. Moreover, Southern Taiwan has experienced a domestic spread of dengue fever over the last 5 years. According to dengue virus type, large spreads occur approximately once every 4 years, and mixed viruses, which result in dengue hemorrhagic fever, can easily occur [40]. This has contributed to a large amount of wasted medical resources and threatened public health. Because domestic sporadic and cluster cases for chikungunya have been observed in Northern Taiwan and chikungunya is spread by the same vector mosquitoes as those that spread dengue fever, epidemic-control specialists of health departments and public-health experts have expressed concerns. Convenient and frequent north–south travel in Taiwan might influence chikungunya and dengue infections and pose challenges for epidemic control. In consideration of this serious public-health challenge, the health department in Taiwan should formulate new control measures in response to concurrent domestic infections of dengue fever and chikungunya as early as possible to prevent and control the diseases.

This study has two limitations. First, the Taiwan CDC’s TNIDSS (infectious disease statistics) includes only basic epidemiological data of patients with chikungunya and provides no clinical data. Therefore, this study could not compare clinical data between patients in terms of their differences or trends. Second, data provided on TNIDSS contain no information about the genotypes or strains of the chikungunya virus. Accordingly, this study could not analyze (1) the type of chikungunya virus strain that spread to Taiwan or (2) the affinity between virus strains in Taiwan and other countries. This study has the advantage of using diverse data provided by Taiwan’s public sector on its online platform (including the initial version of platform). This open platform has stored all historical data, which researchers can use to conduct statistical analyses or create academic value. Such data are worth exploring to expand the monitoring of infectious diseases and their characteristics, thereby continually increasing the capacity of scientific research.

## 5. Conclusions

This study was the first in Taiwan to analyze the epidemiological characteristics and trends of imported and domestic cases of chikungunya from 2007 to 2019. On the basis of data from the Taiwan CDC, this study indicated that one domestic sporadic infection and 20 cases of cluster infection occurred. Moreover, domestic cluster infections of chikungunya in Taiwan exhibited significant differences in age (patients aged 60–69 accounted for the largest proportion), month of confirmation (domestic confirmed cases increased in the month after imported cases increased), and place of residence (New Taipei City accounted for the largest proportion). This information will be useful for policy makers and clinical experts in directing prevention and control measures regarding the chikungunya virus, which causes illness among the Taiwanese population. This study highlights the importance of longitudinal and geographically extended studies to understand the implications of zoonotic disease transmission in the Taiwanese population. Critical data were identified to inform future surveillance and research efforts in Taiwan.

## Figures and Tables

**Table 1:** Epidemiological features by examining chikungunya in domestic and imported cases.

Parameters	Domestic Cases	Imported Cases	*p* Value
2007/1~2019/6	0	119	N/A
2019/7~2019/10	21	79	N/A
Gender			
Male	6	28	0.555
Female	15	51	
Age			
<10 years	0	2	N/A
10–19 years	0	3	N/A
20–29 years	1	24	<0.001
30–39 years	2	9	
40–49 years	2	19	
50–59 years	4	13	
60–69 years	9	8	
≥70 years	3	1	
month/year			
7/2019	1	19	0.005
8/2019	8	37	
9/2019	12	16	
10/2019	0	7	N/A
Cities of Residence			
Taipei City	1	20	0.001
New Taipei City	20	23	
Others	0	36	N/A

N/A: Not applicable.
